# Supplementary material for: Structural basis of ABCF-mediated resistance to pleuromutilin, lincosamide, and streptogramin A antibiotics in Gram-positive pathogens
Source: Nat Commun. 2021 Jun 11;12:3577. doi: 10.1038/s41467-021-23753-1 (PMC8196190; doi:10.1038/s41467-021-23753-1)
Supplement: Supplementary file 1 — Supplementary Information [file 41467_2021_23753_MOESM1_ESM.pdf]

SUPPLEMENTARY DATA FOR

Structural basis of ABCF-mediated resistance to pleuromutilin, lincosamide, and streptogramin A antibiotics in Gram-positive pathogens

Caillan Crowe-McAuliffe[1,#], Victoriia Murina[2,3,#], Kathryn Jane Turnbull[2,3], Marje Kasari[4] Merianne Mohamad[5], Christine Polte[1], Hiraku Takada[2,3], Karolis Vaitkevicius[2,3], Jörgen Johansson[2,3], Zoya Ignatova[1], Gemma C. Atkinson[2], Alex J. O'Neill[5], Vasili Hauryliuk[2,3,4,6,*], Daniel N. Wilson[1,*]

[1] Institute for Biochemistry and Molecular Biology, University of Hamburg, Martin-Luther-King-Platz 6, 20146 Hamburg, Germany.

[2] Department of Molecular Biology, Umeå University, 90187 Umeå, Sweden.

[3] Laboratory for Molecular Infection Medicine Sweden (MIMS), Umeå University, 90187 Umeå, Sweden.

[4] University of Tartu, Institute of Technology, 50411 Tartu, Estonia.

[5] Astbury Centre for Structural Molecular Biology, School of Molecular & Cellular Biology, Faculty of Biological Sciences, University of Leeds, Leeds LS2 9JT, UK.

[6] Department of Experimental Medical Science, Lund University, 221 00 Lund, Sweden.

[#] These authors contributed equally.

*Correspondence to: Daniel.Wilson@chemie.uni-hamburg.de, vasili.hauryliuk@med.lu.se.

Supplementary Figures

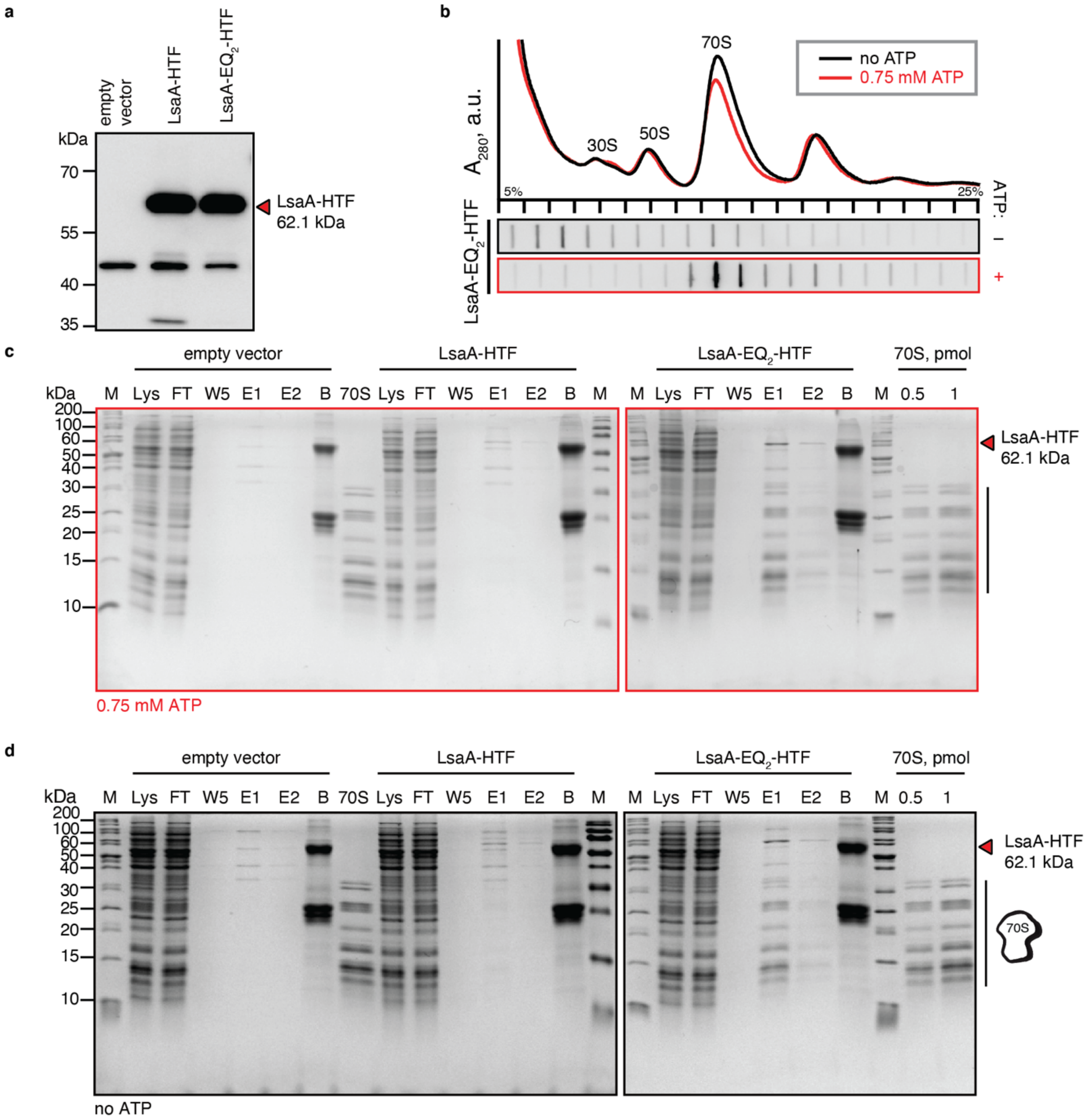

Figure S1. Characterization of *E. faecalis* LsaA interactions with ribosomes and preparation of samples for cryo-EM. a, b Polysome profiles and immunoblot analyses of C-terminally His_6-TEV-$FLAG_3$-tagged (HTF) ATPase-deficient (EQ_2) LsaA-EQ_2 ectopically expressed in Δ*lsaA E. faecalis* TX5332. **a** Specificity of α-$FLAG_3$ detection assessed by probing whole-lysate samples resolved on SDS-PAGE. The immunoprecipitation was repeated at least once. **b** Sucrose gradient centrifugation followed by slot-plot detection. Experiments were performed both in the presence or absence of 0.75 mM ATP in gradients. **c, d** Affinity purification of wild-type and EQ_2 *E. faecalis* LsaA-HTF ectopically expressed in TX5332 *E. faecalis*. Pull-down experiments were performed either in the presence **c** or absence **d** of 0.75 mM ATP using clarified lysates of *E. faecalis* either transformed with empty pCIE vector (background control), expressing *E. faecalis* LsaA-HTF (VHp100) or

expressing *E. faecalis* LsaA-EQ_2-HTF (VHp149). Samples: M: molecular weight marker; Lys: 2 µL of clarified lysate, FT: 2 µL of flow-through; W5: 10 µL of last wash before specific elution; E1: 10 µL of the first elution with $FLAG_3$ peptide; E2: 10 µL of the second elution with $FLAG_3$ peptide; B: 10 µL of SDS-treated post-elution anti-FLAG beads; 70S: purified *E. faecalis* 70S ribosomes. The samples were resolved on 15% SDS-PAGE gel. The 0.75 mM ATP *E. faecalis* LsaA-EQ_2-HTF pulldown sample was used for further cryo-EM and tRNA array analysis. Source data are provided as a Source Data file.

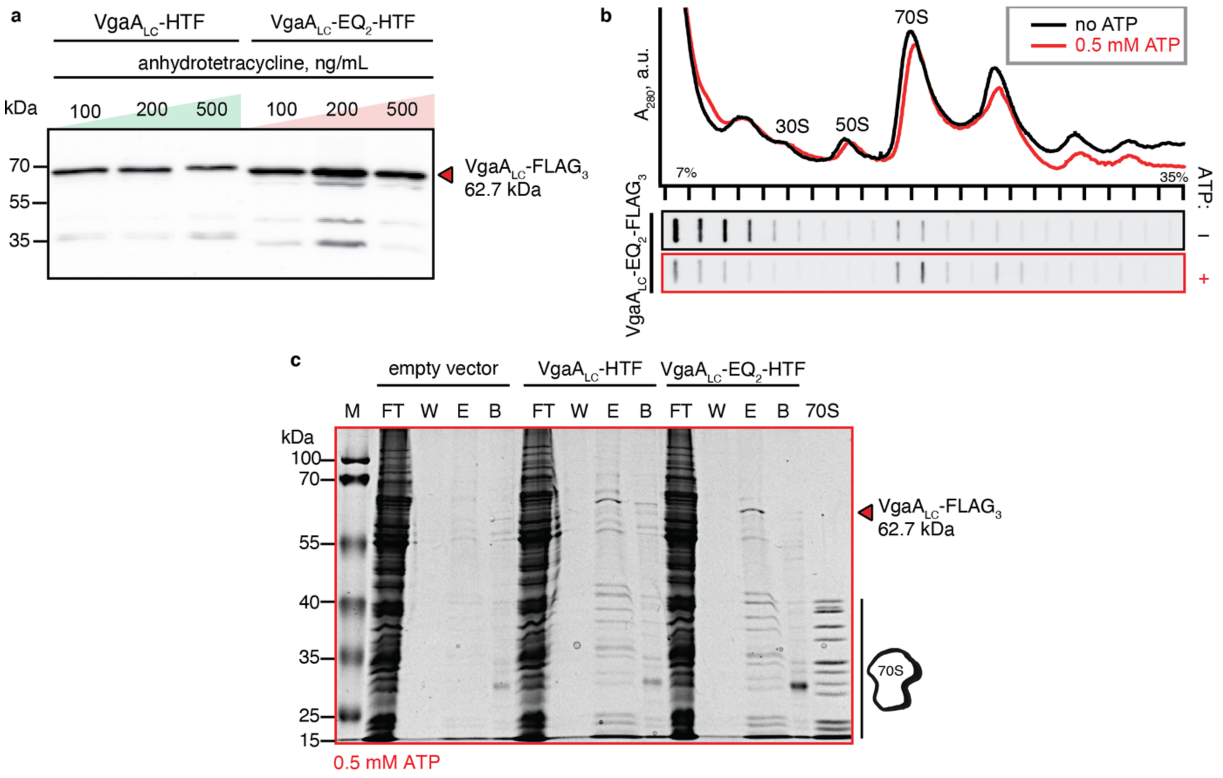

Figure S2. Characterization of *S. haemolyticus* VgaA$_{LC}$ interactions with ribosomes and preparation of samples for cryo-EM reconstructions. a Specificity of α-FLAG$_3$ detection assessed by probing whole-lysate samples resolved on SDS-PAGE. The immunoprecipitation was repeated at least once. **b** Polysome profiles and immunoblot analyses of FLAG$_3$-tagged *S. haemolyticus* VgaA$_{LC}$-EQ$_2$ ectopically expressed in wild-type SH-1000 *S. aureus*. Experiments were performed both in the presence or absence of 0.5 mM ATP in gradients **c** Affinity purification of wild-type and EQ$_2$ *S. haemolyticus* VgaA$_{LC}$-FLAG$_3$ ectopically expressed in SH-1000 *S. aureus*. Immunoprecipitations were performed in the presence of 0.5 mM ATP and the samples were resolved on a 15% polyacrylamide gel by SDS-PAGE. Samples: M: 2 µL of molecular weight marker; FT: 2 µL of flow-through, W: 10 µL of last wash before specific elution; E: 10 µL of elution with FLAG$_3$ peptide; B: 2 µL of SDS-treated post-elution anti-FLAG beads; 70S: 1 pmol of purified *S. aureus* 70S ribosomes. The 0.5 mM ATP *S. haemolyticus* VgaA$_{LC}$-EQ$_2$-HTF pulldown sample was used for cryo-EM reconstructions. Source data are provided as a Source Data file.

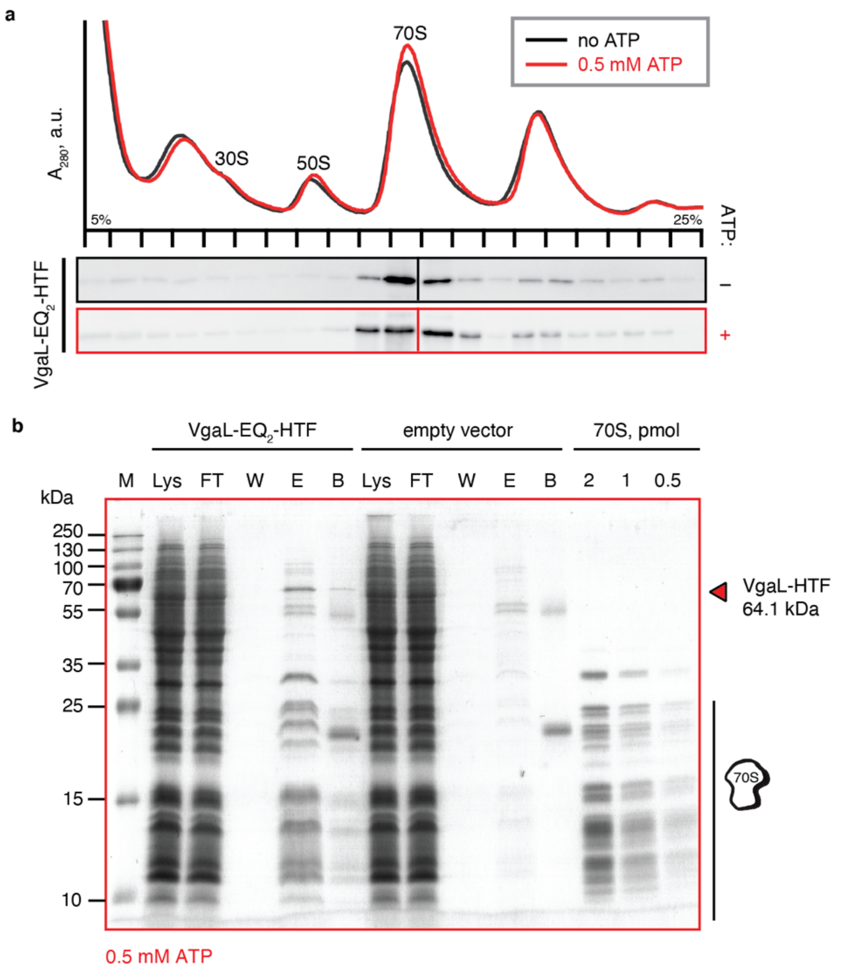

Supplementary Figure 3. Characterization of *L. monocytogenes* VgaL (Lmo0919) interactions with ribosomes and preparation of samples for cryo-EM reconstructions. (**a**) Polysome profiles and immunoblot analyses of HTF-tagged *L. monocytogenes* VgaL-EQ$_2$ (Lmo0919-EQ$_2$) ectopically expressed in wild-type EGD-e *L. monocytogenes*. Experiments were performed both in the presence or absence of 0.5 mM ATP in gradients. Note that all the samples derive from the same experiment and that gels and blots were processed in parallel. The immunoprecipitation was repeated at least once. (**b**) Affinity purification of *L. monocytogenes* VgaL-EQ$_2$ ectopically expressed in EGD-e *L. monocytogenes*. Pull-down experiments were performed in the presence of 0.5 mM ATP using clarified lysates of *L. monocytogenes* transformed with empty integrative pIMK3 vector (background control), expressing VgaL-HTF (VHp692) or expressing VgaL-EQ$_2$-HTF (VHp149). Samples: M: 2 µL of molecular weight marker; FT: 2 µL of flow-through; W: 10 µL of last wash before specific elution; E: 10 µL of elution with FLAG$_3$ peptide; B: 2 µL of SDS-treated post-elution anti-FLAG beads; 70S: purified *B. subtilis* 70S ribosomes, the samples were resolved on 15 % SDS-PAGE gel. The 0.5 mM ATP *L. monocytogenes* VgaL-EQ$_2$-HTF pulldown sample was used for cryo-EM reconstructions. Source data are provided as a Source Data file.

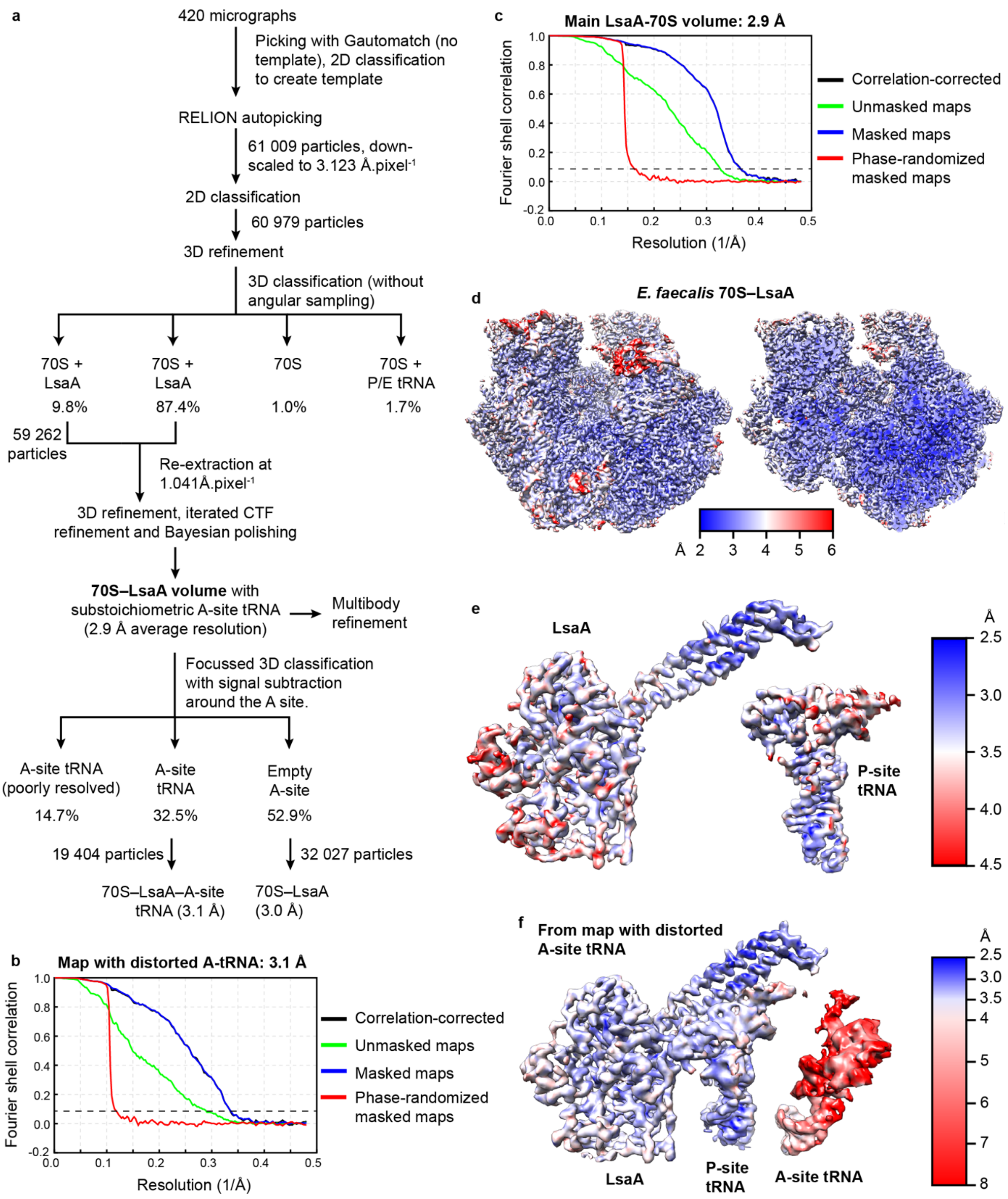

Supplementary Figure 4. Processing of the cryo-EM data of LsaA-70S complex. a Processing scheme for the LsaA-70S complex, yielding two subpopulations of LsaA-70S complexes with and without A-site tRNA. **b,c** Fourier Shell Correlation (FSC) curves of the LsaA-70S **b** with A-tRNA and **c** without A-tRNA with a dashed line at 0.143 indicating average resolutions of 3.1 Å and 2.9 Å, respectively. **d** Overview (left) and transverse section (right) of the cryo-EM map of the LsaA-70S (without A-tRNA) coloured according to local resolution. **e** Isolated density of LsaA (left) and P-site tRNA (right) from **d**. **f** Isolated density of LsaA, P-site and A-site tRNA from the LsaA-70S map (with A-tRNA) coloured according to local resolution.

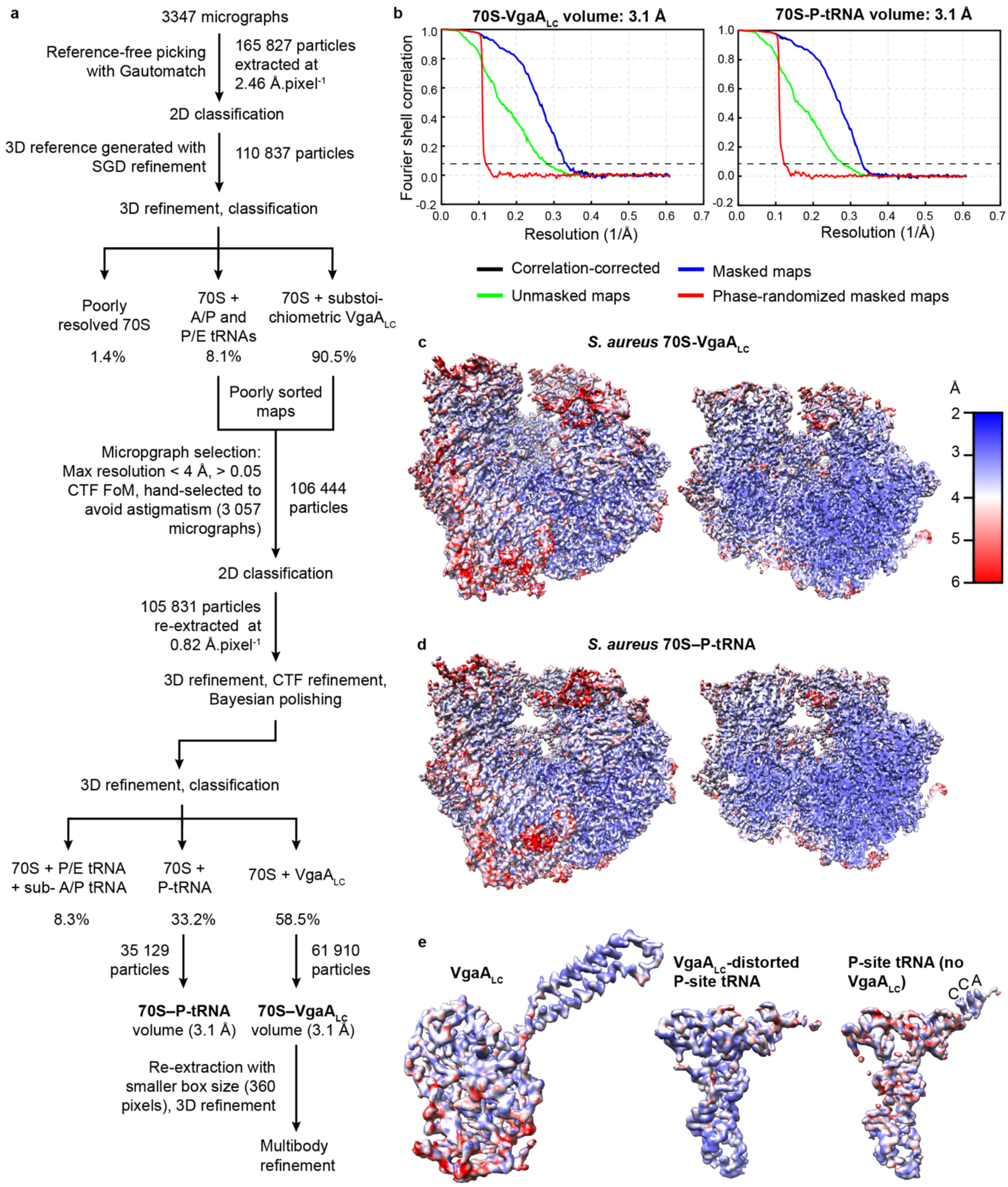

Supplementary Figure 5. Processing of the cryo-EM data of VgaA$_{LC}$-70S complex. (**A**) Processing scheme for the LsaA-70S complex, yielding a VgaA$_{LC}$-70S and 70S-P-tRNA complex without VgaA$_{LC}$. (**B**) Fourier Shell Correlation (FSC) curves of the VgaA$_{LC}$-70S and 70S-P-tRNA complexes with a dashed line at 0.143 indicating average resolutions of 3.1 Å. (**C, D**) Overview (left) and transverse section (right) of the cryo-EM map of the (**C**) VgaA$_{LC}$-70S and (**D**) 70S-P-tRNA complexes coloured according to local resolution. (**E**) Isolated density of VgaA$_{LC}$ (left) and P-site tRNA (right) from the VgaA$_{LC}$-70S complex, and the P-site-tRNA from the 70S–P-tRNA complex, coloured according to local resolution.

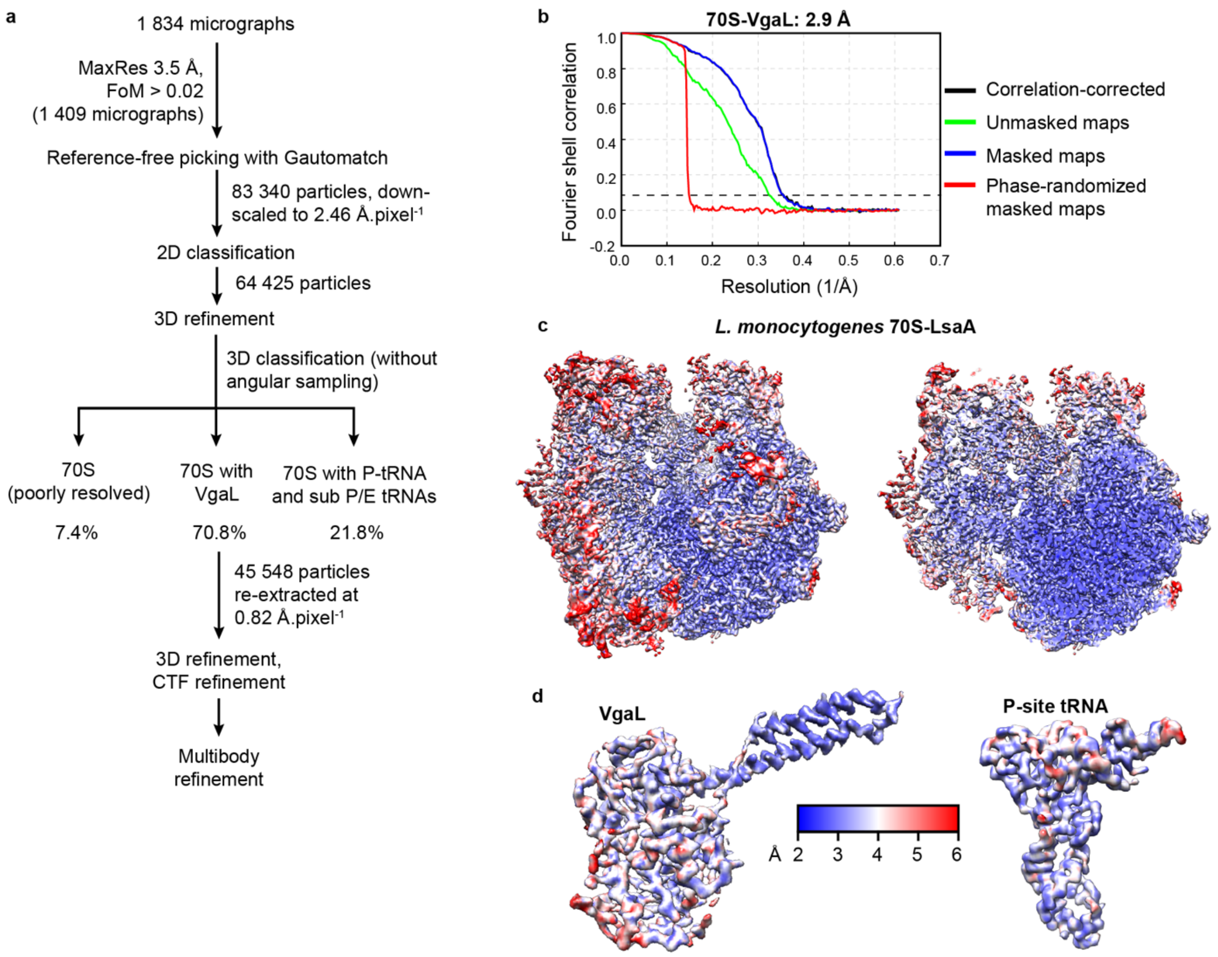

Supplementary Figure 6. Processing of the cryo-EM data of VgaL-70S complex. (**A**) Processing scheme for the VgaL-70S complex. (**B**) Fourier Shell Correlation (FSC) curves of the VgaL-70S complex with a dashed line at 0.143 indicating average resolutions of 2.9 Å. (**C**) Overview (left) and transverse section (right) of the cryo-EM map of the VgaL-70S complex coloured according to local resolution. (**D**) Isolated density of VgaL (left) and P-site tRNA (right) from the VgaL-70S complex coloured according to local resolution.

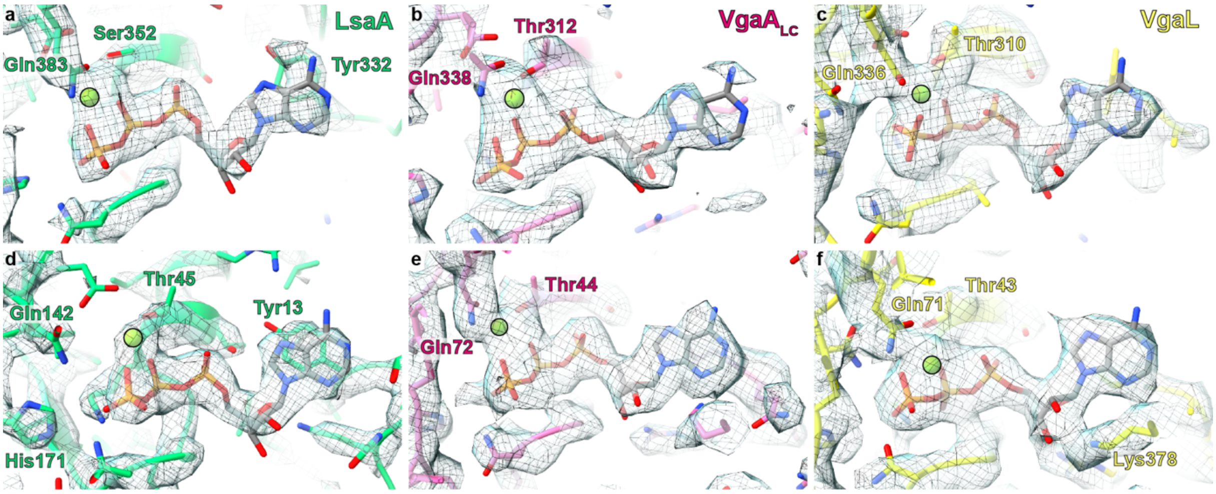

Supplementary Figure 7. ATP in the ARE-bound 70S structures. Model and density surrounding the outermost ATP (site 1) bound by LsaA (**a**), VgaA$_{LC}$ (**b**), and VgaL (**c**) viewed from the direction of the signature sequence of NBD2 (not shown). A black outline highlights a putative magnesium ion. **d–f**, as for **a–c** except for the innermost nucleotide-binding site (site 1) viewed from the direction of the signature sequence of NBD1 (not shown). Density from post-processed maps is shown.

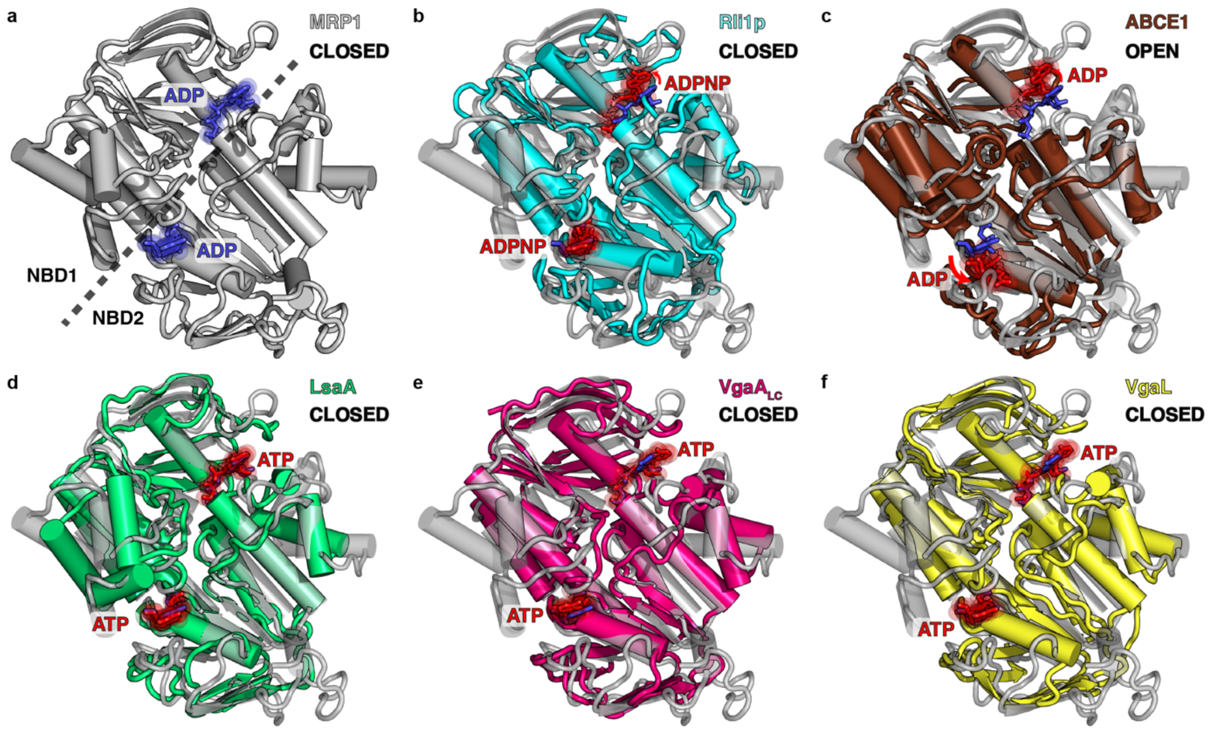

Supplementary Figure 8. LsaA, VgaA$_{LC}$ and VgaL NBDs exhibit a closed conformation. **a** The closed conformation of the multidrug transporter MRP1 (grey) with bound ADP molecules (blue, PDB 6BHU)[1]. **b,c** Alignment (based on NBD1) and superimposition of the closed conformation of MRP1 from **a** with the ABC domains of **b** Rli1p (cyan) in closed conformation with bound ADPNP (red, PDB 5LL6)[2], **c** ABCE1 (brown) in open conformation with bound ADP (red, PDB 3J15)[3], and with **d–f** closed ARE-ABCF NBD conformations with bound ATP (red) for **d** LsaA (green), **e** VgaA$_{LC}$ (magenta) and **f** VgaL (yellow).

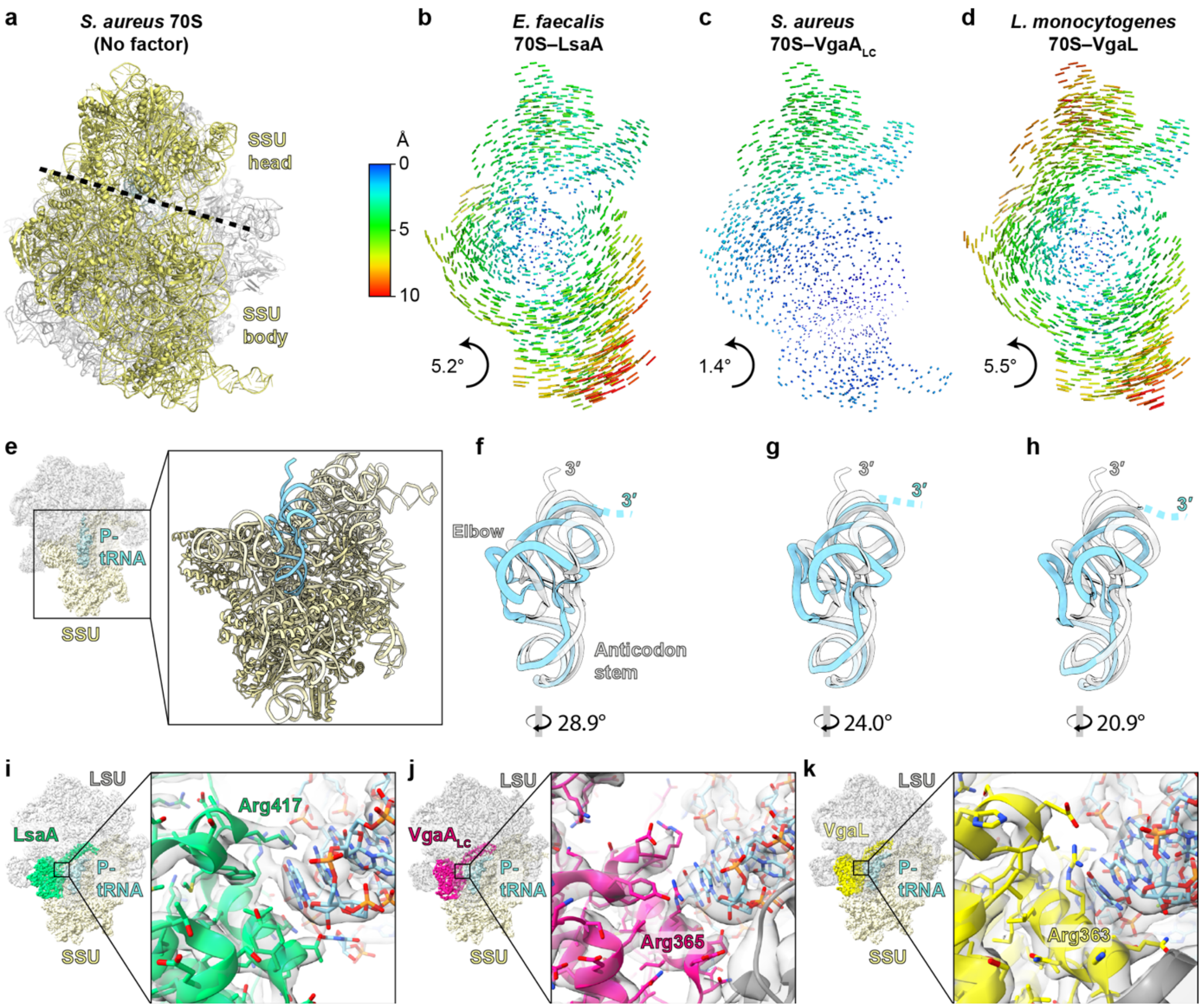

Supplementary Figure 9. Small subunit and P-tRNA rotation in the ARE-bound 70S structures. a *S. aureus* 70S ribosome with P-tRNA and no ARE viewed from the small subunit-solvent interface. **b–d** comparison of the small subunit in the non-rotated state (shown in **a**) and the small subunit from the ARE-bound structures. Movements between residues are represented as lines coloured according to the distance moved. **b**, *E. faecalis* LsaA-70S; **c**, *S. aureus* VgaA$_{LC}$-70S; **d**, *L. monocytogenes* VgaL-70S. **e** overview of *S. aureus* 70S ribosome with P-tRNA with inset showing position of P-tRNA on the small subunit. **f–h** the canonical, non-rotated P-tRNA from **e** (light grey) compared with P-tRNAs from ARE-bound structures (blue), with the rotation quantified below. **f**, *E. faecalis* LsaA-70S; **g**, *S. aureus* VgaA$_{LC}$-70S; **h**, *L. monocytogenes* VgaL-70S. A dashed line represents the likely path of the 3’ CCA end, which is not included in the models, for the tRNAs from the ARE-bound 70S structures. **i–k**, overviews with insets showing the site of interaction between each ARE and the P-tRNA elbow. **i**, *E. faecalis* LsaA-70S; **j**, *S. aureus* VgaA$_{LC}$-70S; **k**, *L. monocytogenes* VgaL-70S.

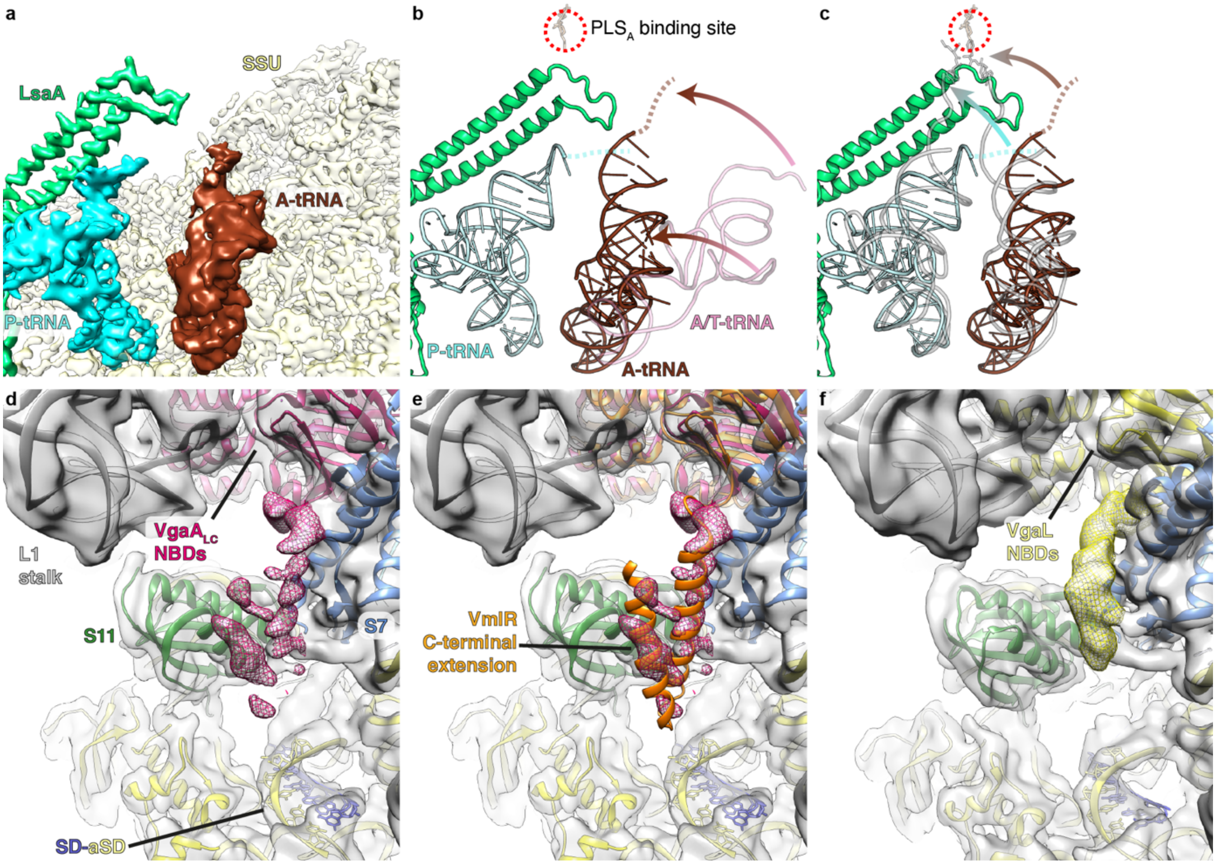

Supplementary Figure 10. Presence of A-site tRNA in the LsaA-70S complex. a Cryo-EM map density for LsaA (green), P-site tRNA (cyan) and A-site tRNA (brown) in the LsaA-70S complex with A-site tRNA. Density for small subunit (yellow) is shown for reference. Density for the large subunit is not shown. **b** The same view as A, except with molecular models. The brown dashed line indicates a likely path for the 3′ CCA end of the distorted A-tRNA. A pre-accommodation A/T tRNA (pink, PDB 4V5L)[4] is superimposed. The position of the lincomycin binding site (red dotted circle) is shown for comparison (PDB 5HKV)[5]. **c** Similar to **b** except with classical accommodated A- and P-site tRNAs from pre-attack state superimposed (both grey, PDB 1VY4)[6]. **d–f** Cryo-EM map (grey) with molecular model for **e–f** VgaA$_{LC}$-70S complex, and (**f**) VgaL-70S complex, showing density for L1 stalk (grey) on the large subunit, and ribosomal proteins S7 (blue), S11 (green) as well as the SD–anti-SD helix on the small subunit (yellow). In **d** and **e**, density for the C-terminal extension (CTE) of VgaA$_{LC}$ (magenta mesh) is fragmented, and in **e** fitted with the model of the CTE from VmlR (orange, PDB 6HA8)[7] based on alignment of the NBDs. In **f**, density for the C-terminal extension (CTE) of VgaL (yellow mesh) also reaches between the S7–S11 cleft and is consistent with an α-helical conformation, but appears to be distinct from VmlR and VgaA$_{LC}$ and could not be modelled at this resolution.

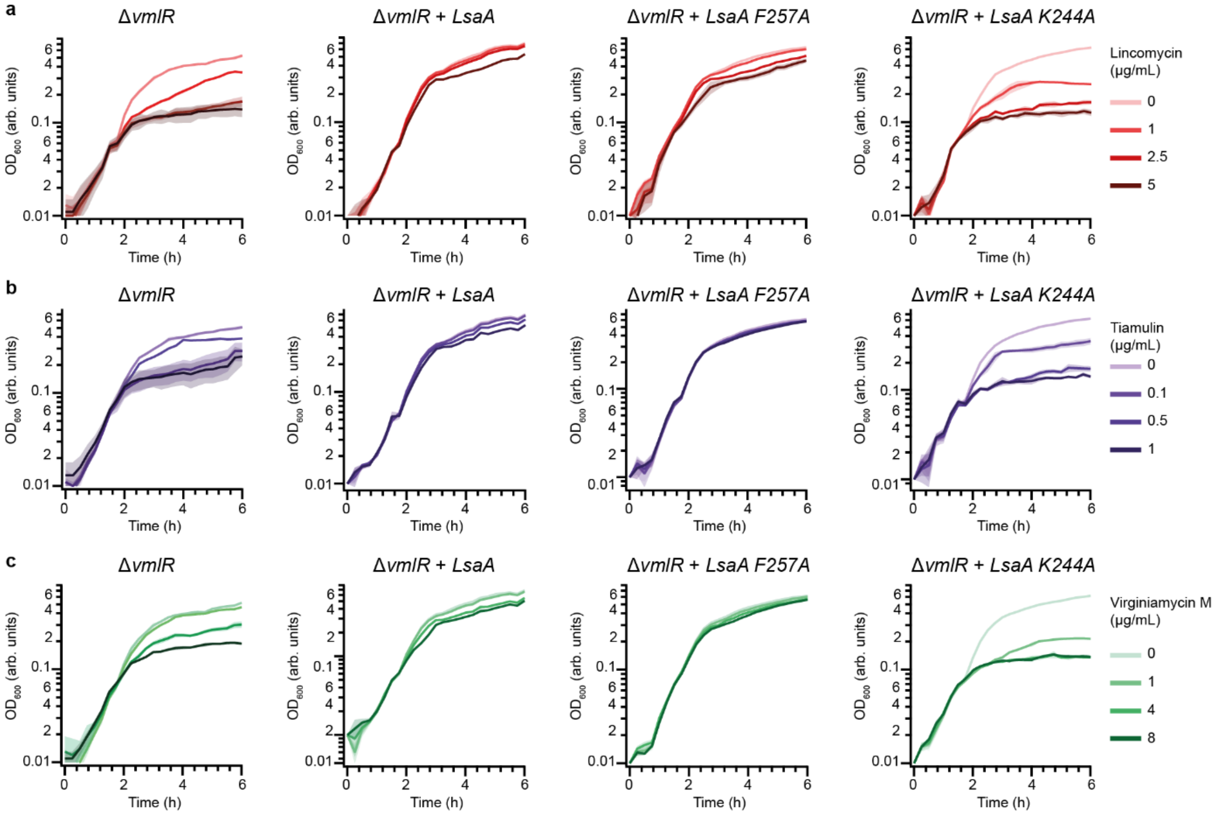

Supplementary Figure 11. Effect of amino acid substitutions in ARD on antibiotic resistance in LsaA. Growth of *B. subtilis* Δ*vmlR* expressing the indicated LsaA variants over time in the presence of lincomycin **a**, tiamulin **b**, and virginiamycin M **c**. *B. subtilis* strains (VHB109, 168 and 169) were grown in LB media with 1 mM IPTG at 37 °C with medium shaking. At the 90 minutes time point (OD_{600} ≈ 0.1) antibiotics were added to the final concentrations as indicated on the figure. The mean is shown as a line with the SD of three biological replicates is indicated with pale shading. The leftmost panels, showing susceptibility to the antibiotics in the base Δ*vmlR* strain, are the same as used in Crowe-McAuliffe *et al.*, 2018[7]. Source data are provided as a Source Data file.

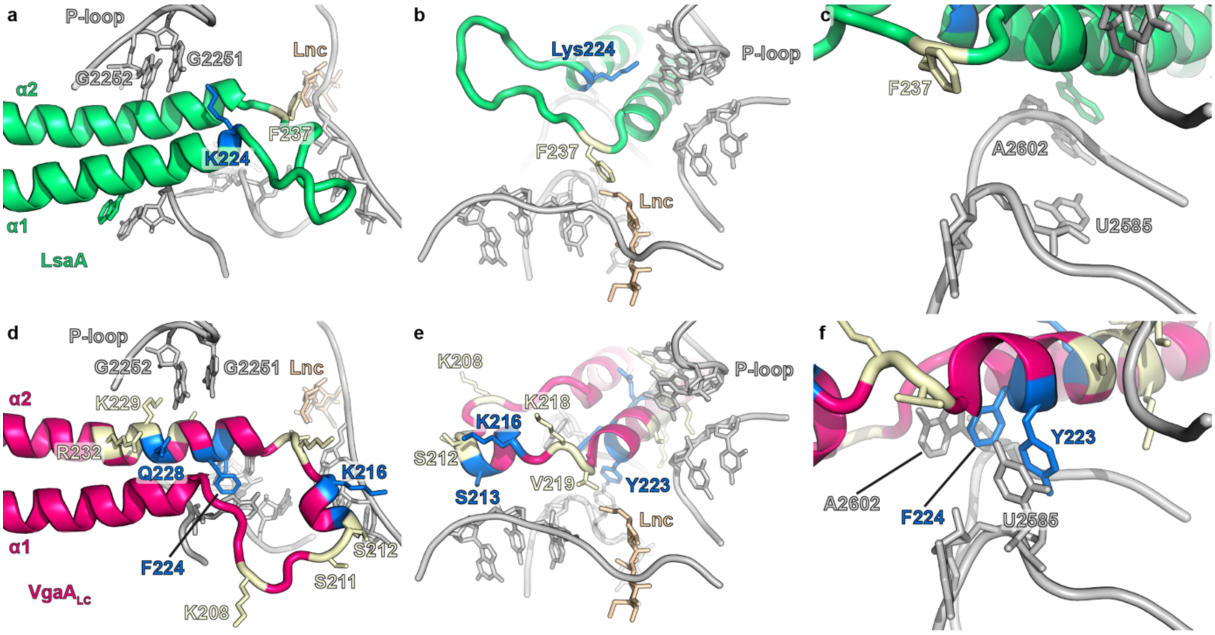

Supplementary Figure 12. Visualisation of tested mutations in $VgaA_{LC}$ and LsaA. Residues in blue reduced antibiotic resistance when mutated to alanine, and residues in yellow did not affect antibiotic resistance when mutated to alanine. **a–c**, three views of the LsaA ARD with selected *E. faecalis* 23S 23S rRNA nucleotides shown. **d–f**, three views of the $VgaA_{LC}$ ARD with selected *S. aureus* 23S 23S rRNA nucleotides shown. See also Tables S1 and S2.

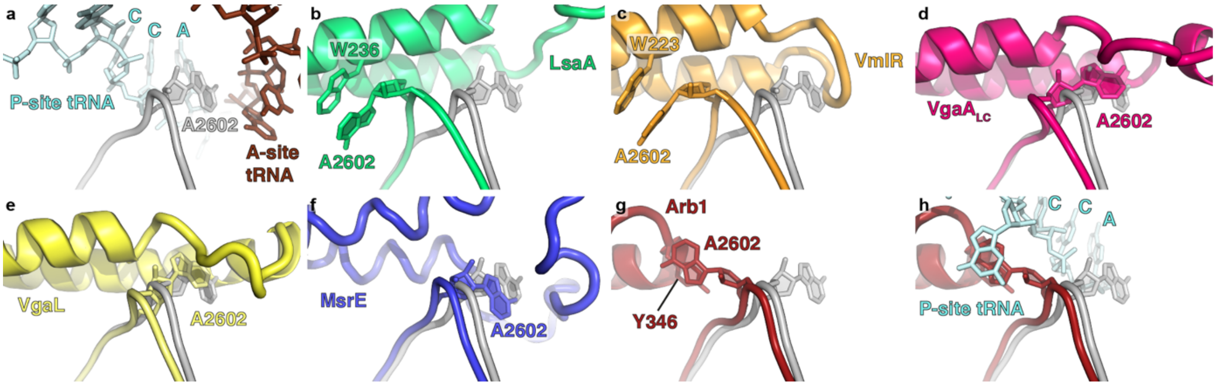

Supplementary Figure 13. Comparison of A2602 position between ribosomes with and without bound AREs. a A2602 with accommodated A- and P-site tRNAs in the 'pre-attack' state (PDB 1VY4)[6] **b** Conformation of A2602 with bound LsaA with 23S rRNA from **a**. **c–g** Similar to **b**, except for VmlR (PDB 6HA8)[7], VgaA$_{LC}$, VgaL, MsrE (PDB 5ZLU)[8], and Arb1 (PDB 6R84)[9].

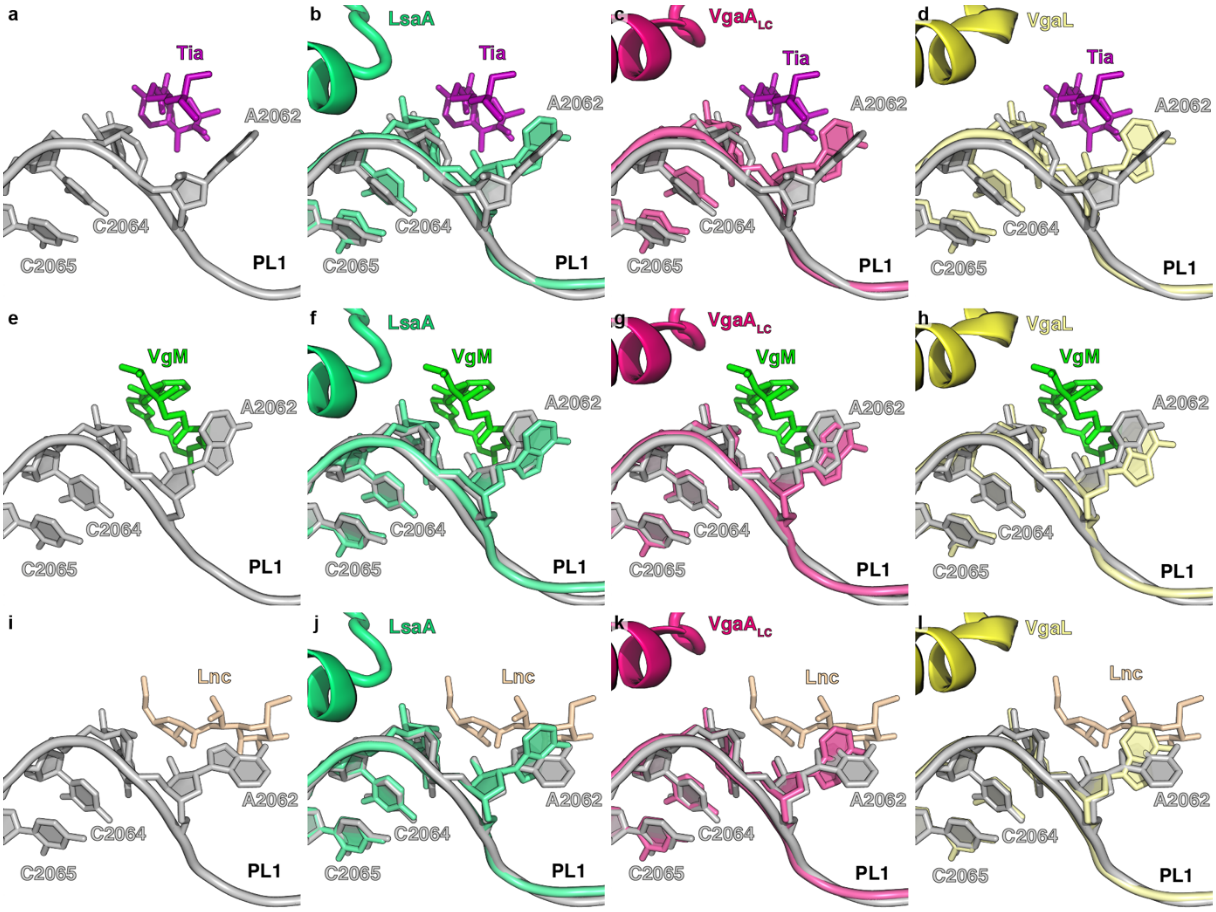

Supplementary Figure 14. PTC loop 1 in the presence of PLS$_A$ antibiotics and AREs. a–d The conformation of selected nucleotides from PL1 at the PLS$_A$ binding site in the presence of tiamulin (Tia, purple, PDB 1XBP)[10] and LsaA (**b**, green nucleotides), VgaA$_{LC}$ (**c**, pink nucleotides), and VgaL (**d**, yellow nucleotides) **e–h** Same as **a–d** but with virginiamycin M (VgM, green, PDB 4U25)[11] instead of tiamulin. **i–l** as for **a–d** but with lincomycin (Lnc, tan, PDB 5HKV)[5] instead of tiamulin.

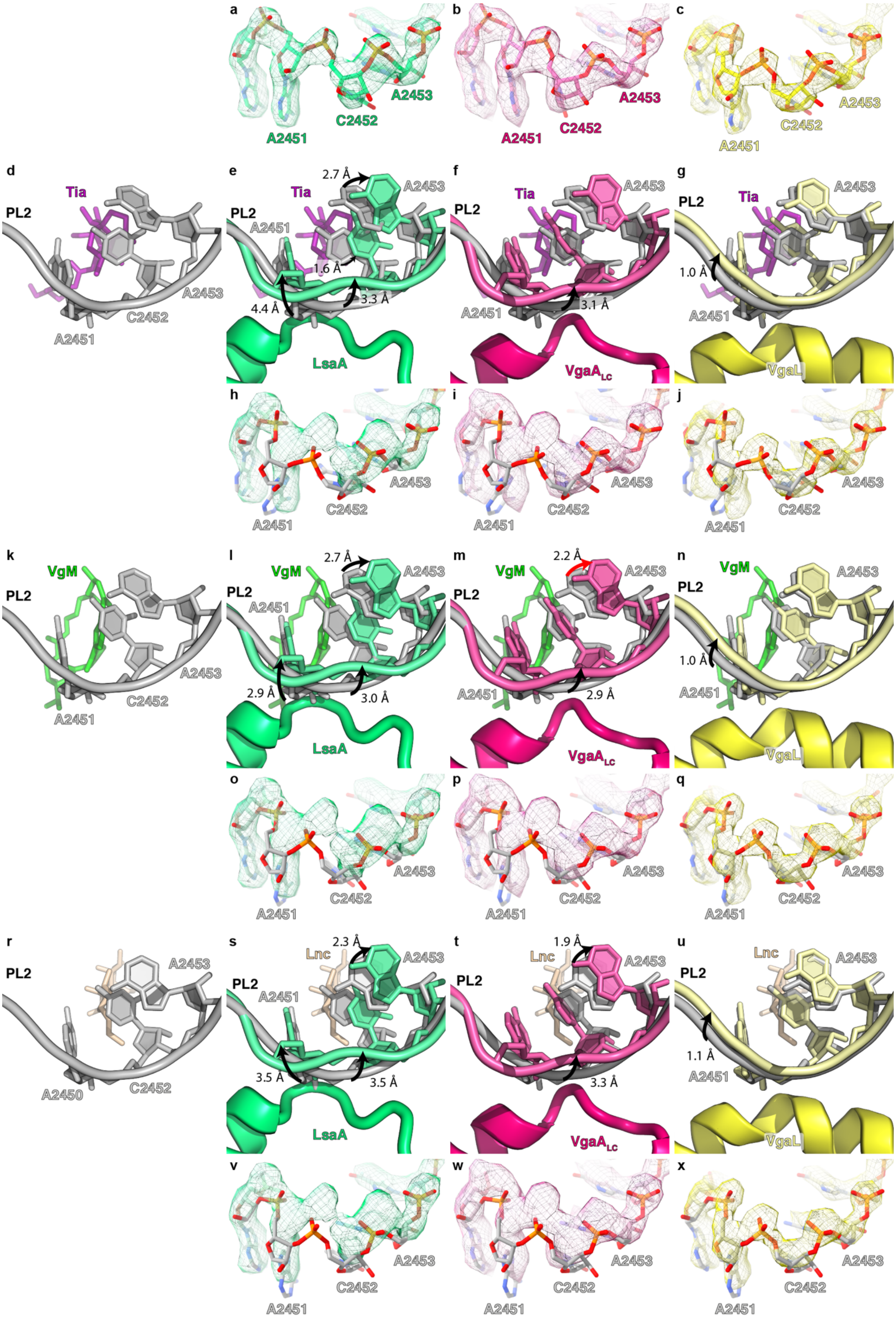
a
A2451
C2452
A2453
b
A2451
C2452
A2453
c
A2451
C2452
A2453
d
PL2
Tia
A2451
C2452
A2453
e
PL2
Tia
A2451
2.7 Å
1.6 Å
4.4 Å
3.3 Å
A2453
LsaA
f
PL2
Tia
A2451
3.1 Å
A2453
VgaA$_{LC}$
g
PL2
Tia
1.0 Å
A2451
A2453
VgaL
h
A2451
C2452
A2453
i
A2451
C2452
A2453
j
A2451
C2452
A2453
k
PL2
VgM
A2451
C2452
A2453
l
PL2
VgM
A2451
2.7 Å
2.9 Å
3.0 Å
A2453
LsaA
m
PL2
VgM
2.2 Å
A2451
2.9 Å
A2453
VgaA$_{LC}$
n
PL2
VgM
1.0 Å
A2451
A2453
VgaL
o
A2451
C2452
A2453
p
A2451
C2452
A2453
q
A2451
C2452
A2453
r
PL2
A2450
C2452
A2453
s
PL2
Lnc
A2451
2.3 Å
3.5 Å
3.5 Å
A2453
LsaA
t
PL2
Lnc
1.9 Å
3.3 Å
A2453
VgaA$_{LC}$
u
PL2
Lnc
1.1 Å
A2451
A2453
VgaL
v
A2451
C2452
A2453
w
A2451
C2452
A2453
x
A2451
C2452
A2453

Supplementary Figure 15. PTC loop 2 in the presence of PLS_A antibiotics and AREs. a–c Density and model of selected nucleotides from PL2 in the ARE-bound form.
d–g Selected nucleotides from PL2 at the PLS_A binding site in the presence of tiamulin (Tia, purple, PDB 1XBP)[10] and LsaA (**e**, green nucleotides), $VgaA_{LC}$ (**f**, pink nucleotides), and VgaL (**g**, yellow nucleotides). **h–j** Density of selected nucleotides from PL2 in the ARE-bound form (same as **a–c**) with model from the tiamulin-bound state superimposed.
k–n Selected nucleotides from PL2 at the PLS_A binding site in the presence of virginiamycin M (VgM, green, PDB 4U25)[11] and LsaA (**l**, green nucleotides), $VgaA_{LC}$ (**m**, pink nucleotides), and VgaL (**n**, yellow nucleotides). **o–q** Density of selected nucleotides from PL2 in the ARE-bound form (same as **a–c**) with model from the virginiamycin M-bound state superimposed.
r–u Selected nucleotides from PL2 at the PLS_A binding site in the presence of lincomycin (Lnc, tan, PDB 5HKV)[5] and LsaA (**s**, green nucleotides), $VgaA_{LC}$ (**t**, pink nucleotides), and VgaL (**u**, yellow nucleotides). **v–x** Density of selected nucleotides from PL2 in the ARE-bound form (same as **a–c**) with model from the lincomycin-bound state superimposed.

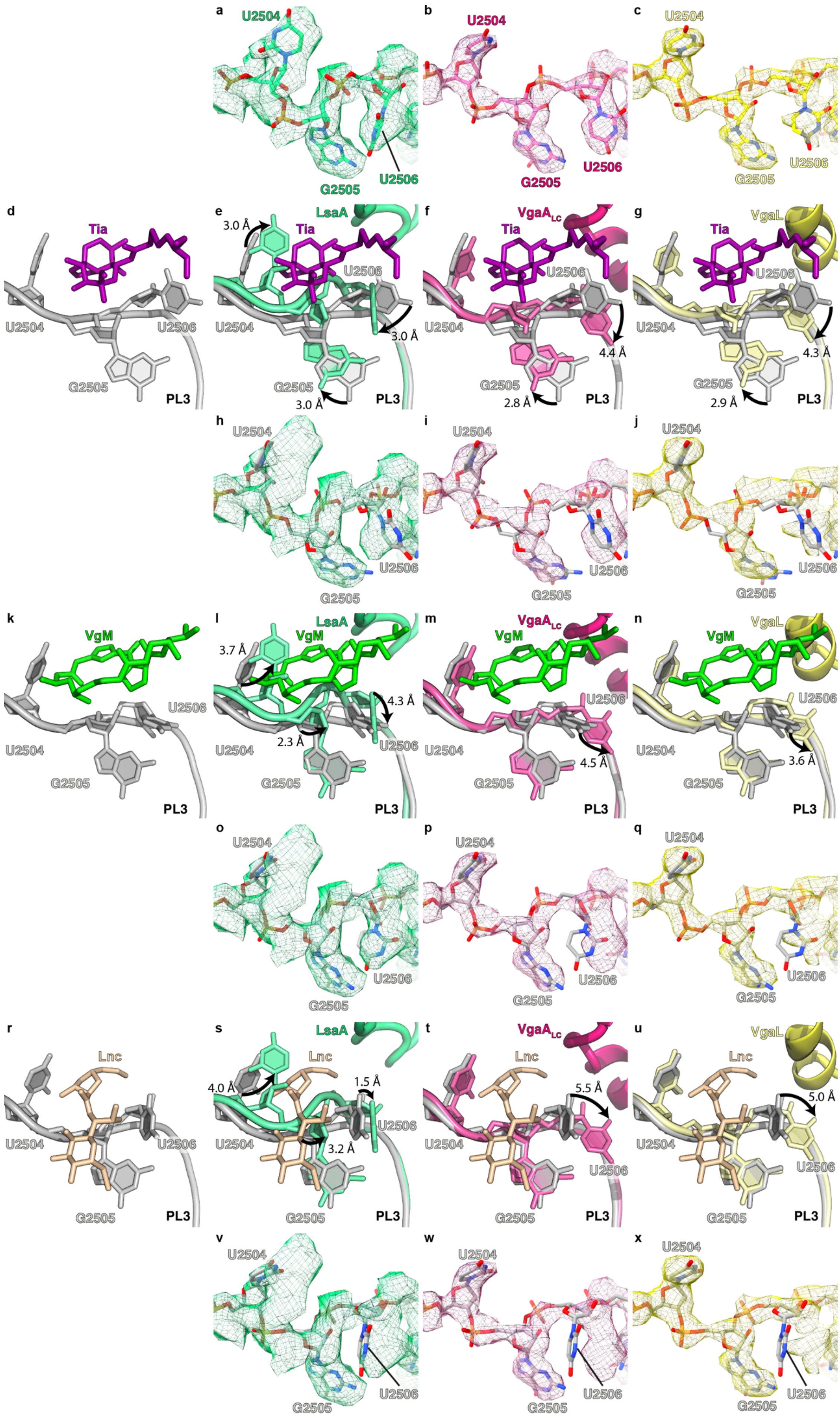
a
U2504
G2505
U2506
b
U2504
G2505
U2506
c
U2504
G2505
U2506
d
Tia
U2504
U2506
G2505
PL3
e
LsaA
Tia
3.0 Å
U2506
U2504
3.0 Å
G2505
3.0 Å
PL3
f
VgaA$_{LC}$
Tia
U2506
U2504
4.4 Å
G2505
2.8 Å
PL3
g
VgaL
Tia
U2506
U2504
4.3 Å
G2505
2.9 Å
PL3
h
U2504
U2506
G2505
i
U2504
U2506
G2505
j
U2504
U2506
G2505
k
VgM
U2506
U2504
G2505
PL3
l
LsaA
VgM
3.7 Å
4.3 Å
2.3 Å
U2504
U2506
G2505
PL3
m
VgaA$_{LC}$
VgM
U2506
U2504
4.5 Å
G2505
PL3
n
VgaL
VgM
U2506
U2504
3.6 Å
G2505
PL3
o
U2504
U2506
G2505
p
U2504
U2506
G2505
q
U2504
U2506
G2505
r
Lnc
U2504
U2506
G2505
PL3
s
LsaA
Lnc
4.0 Å
1.5 Å
U2506
U2504
3.2 Å
G2505
PL3
t
VgaA$_{LC}$
Lnc
5.5 Å
U2504
U2506
G2505
PL3
u
VgaL
Lnc
5.0 Å
U2504
U2506
G2505
PL3
v
U2504
U2506
G2505
w
U2504
U2506
G2505
x
U2504
U2506
G2505

Supplementary Figure 16. PTC loop 3 in the presence of PLS_A antibiotics and AREs. a–c Density and model of selected nucleotides from PL3 in the ARE-bound form.
d–g Selected nucleotides from PL3 at the PLS_A binding site in the presence of tiamulin (Tia, purple, PDB 1XBP)[10] and LsaA (**e**, green nucleotides), $VgaA_{LC}$ (**f**, pink nucleotides), and VgaL (**g**, yellow nucleotides). **h–j** Density of selected nucleotides from PL3 in the ARE-bound form (same as **a–c**) with model from the tiamulin-bound state superimposed.
k–n Selected nucleotides from PL3 at the PLS_A binding site in the presence of virginiamycin M (VgM, green, PDB 4U25)[11] and LsaA (**l**, green nucleotides), $VgaA_{LC}$ (**m**, pink nucleotides), and VgaL (**n**, yellow nucleotides). **o–q** Density of selected nucleotides from PL3 in the ARE-bound form (same as **a–c**) with model from the virginiamycin M-bound state superimposed.
r–u Selected nucleotides from PL3 at the PLS_A binding site in the presence of lincomycin (Lnc, tan, PDB 5HKV)[5] and LsaA (**s**, green nucleotides), $VgaA_{LC}$ (**t**, pink nucleotides), and VgaL (**u**, yellow nucleotides). **v–x** Density of selected nucleotides from PL3 in the ARE-bound form (same as **a–c**) with model from the lincomycin-bound state superimposed.

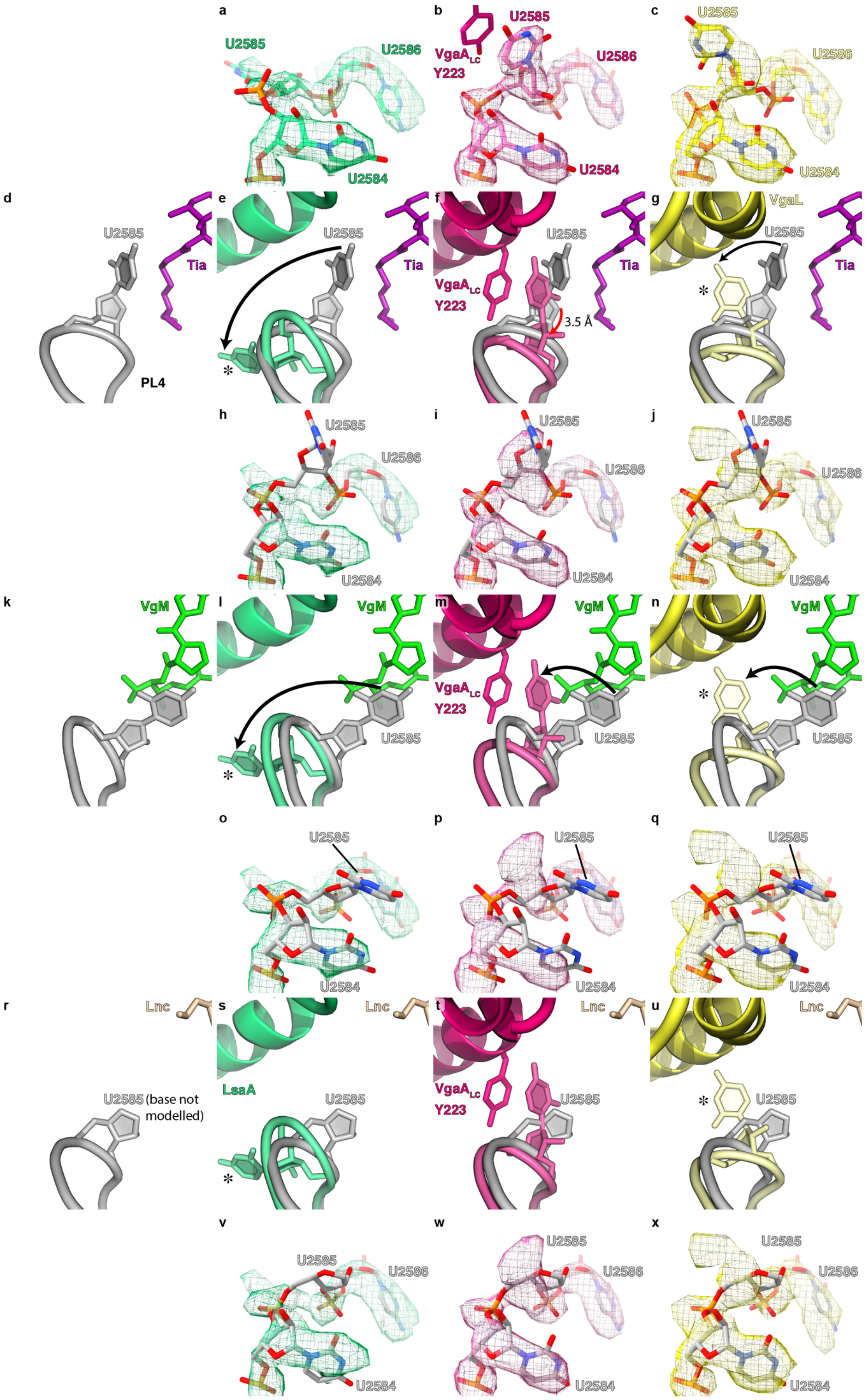

a
U2585
U2586
U2584
b
VgaA$_{LC}$
Y223
U2585
U2586
U2584
c
U2585
U2586
U2584
d
U2585
Tia
PL4
e
U2585
Tia
*
f
U2585
Tia
VgaA$_{LC}$
Y223
3.5 Å
g
VgaL
U2585
Tia
*
h
U2585
U2586
U2584
i
U2585
U2586
U2584
j
U2585
U2586
U2584
k
VgM
l
VgM
U2585
*
m
VgM
VgaA$_{LC}$
Y223
U2585
n
VgM
*
U2585
o
U2585
U2584
p
U2585
U2584
q
U2585
U2584
r
Lnc
U2585 (base not modelled)
s
Lnc
LsaA
U2585
*
t
Lnc
VgaA$_{LC}$
Y223
U2585
u
Lnc
*
U2585
v
U2585
U2586
U2584
w
U2585
U2586
U2584
x
U2585
U2586
U2584

Supplementary Figure 17. PTC loop 4 in the presence of PLS_A antibiotics and AREs. a–c Density and model of selected nucleotides from PL3 in the ARE-bound form.
d–g Selected nucleotides from PL4 at the PLS_A binding site in the presence of tiamulin (Tia, purple, PDB 1XBP)[10] and LsaA (**e**, green nucleotides), $VgaA_{LC}$ (**f**, pink nucleotides), and VgaL (**g**, yellow nucleotides). **h–j** Density of selected nucleotides from PL4 in the ARE-bound form (same as **a–c**) with model from the tiamulin-bound state superimposed.
k–n Selected nucleotides from PL4 at the PLS_A binding site in the presence of virginiamycin M (VgM, green, PDB 4U25)[11] and LsaA (**l**, green nucleotides), $VgaA_{LC}$ (**m**, pink nucleotides), and VgaL (**n**, yellow nucleotides). **o–q** Density of selected nucleotides from PL4 in the ARE-bound form (same as **a–c**) with model from the virginiamycin M-bound state superimposed.
r–u Selected nucleotides from PL4 at the PLS_A binding site in the presence of lincomycin (Lnc, tan, PDB 5HKV)[5] and LsaA (**s**, green nucleotides), $VgaA_{LC}$ (**t**, pink nucleotides), and VgaL (**u**, yellow nucleotides). **v–x** Density of selected nucleotides from PL4 in the ARE-bound form (same as **a–c**) with model from the lincomycin-bound state superimposed.

Table S1. Minimum inhibitory concentrations (MICs) of ribosome-targeting antibiotics against *E. faecalis* expressing LsaA. 5 x 10^5 CFU/mL (OD_{600} approximately 0.0005) of either *E. faecalis* OG1RF, Δ*lsaA* (*lsa::Kan*) strain TX5332 transformed with empty $pCIE_{spec}$ plasmid, or with $pCIE_{spec}$ derivative for expression of LsaA was used to inoculate BHI media supplemented with 2 mg/mL kanamycin to prevent *lsa* revertants, 0.1 mg/mL spectinomycin to maintain the $pCIE_{spec}$ plasmid, 100 ng/mL of cCF10 peptide to induce expression of LsaA as well as increasing concentrations of antibiotics. After 16–20 hours at 37 °C without shaking, the presence or absence of bacterial growth was scored by eye. The MIC values that exceed the empty vector control by at least 4-fold are shown in bold.

		MIC, µg/mL			
antibiotic class	antibiotic	*E. faecalis* OG1RF	*E. faecalis* TX5332 $pCIE_{spec}$	*E. faecalis* TX5332 $pCIE_{spec}$ *lsaA*	*E. faecalis* TX5332 pCIE *lsaA-HTF*
phenicols	chloramphenicol	2-4	2-4	2-4	
	thiamphenicol	4	4	4	
	florfenicol	1	1-2	1-2	
oxazolidinones	linezolid	1	1	1	1
macrolides	erythromycin	1	0.5-1	0.5	0.5
	azithromycin	1-2	0.5-1	0.5-1	
	leucomycin	0.5-1	0.5	0.5-1	
lincosamides	lincomycin	**32**	0.125	**16-32**	**8-16**
	clindamycin	**16-32**	0.0156	**16**	**4-8**
pleuromutilins	tiamulin	**128**	0.0625	**128**	**32-64**
	retapamulin	**>64**	0.0156	**>64**	
streptogramins	virginiamycin M1	**>64**	4	**>128**	
	virginiamycin S1	8	8	8	
tetracyclines	tetracycline	0.5	0.25	0.25	

Table S2. Minimum inhibitory concentrations (MICs) of ribosome-targeting antibiotics against *S. aureus* expressing VgaA$_{LC}$ *S. aureus* strain SH1000, harbouring empty vector pRMC2 or pRMC2 expressing wild-type *vgaA*$_{LC}$ or its mutants. The MIC values that exceed the empty vector control by at least 4-fold are shown in bold.

	MIC, µg/mL				
Construct (mutation)	lincomycin	clindamycin	tiamulin	retapamulin	virginiamycin M1
pRMC2	0.5	0.06	0.5	0.06	2
pRMC2: *vgaA*$_{LC}$	**16**	**2**	**8**	**4**	4
pRMC2: *vgaA*$_{LC}$ ($K_{208}A$)	**16**	**2**	**16**	**8**	4
pRMC2:*vgaA*$_{LC}$ ($S_{211}A$)	**16**	**2**	**16**	**4**	4
pRMC2:*vgaA*$_{LC}$ ($S_{212}A$)	**8**	**2**	**16**	**8**	4
pRMC2:*vgaA*$_{LC}$ ($S_{213}A$)	**2**	0.125	1	0.125	1
pRMC2:*vgaA*$_{LC}$ ($K_{216}A$)	**8**	**0.5**	**4**	**1**	4
pRMC:*vgaA*$_{LC}$ ($K_{218}A$)	**16**	**1**	**16**	**4**	4
pRMC2:*vgaA*$_{LC}$ ($V_{219}A$)	**16**	**1**	**16**	**8**	2
pRMC2:*vgaA*$_{LC}$ ($Y_{223}A$)	**2**	0.125	1	0.125	1
pRMC2:*vgaA*$_{LC}$ ($F_{224}A$)	0.5	0.06	0.25	0.06	1
pRMC2:*vgaA*$_{LC}$ ($S_{226}A$)	**16**	**2**	**16**	**8**	4
pRMC2:*vgaA*$_{LC}$ ($K_{227}A$)	**4**	**0.25**	1	0.125	2
pRMC2:*vgaA*$_{LC}$ ($Q_{228}A$)	**8**	**2**	**8**	**2**	2
pRMC2:*vgaA*$_{LC}$ ($K_{229}A$)	**16**	**2**	**8**	**4**	4
pRMC2:*vgaA*$_{LC}$ ($K_{230}A$)	**16**	**2**	**8**	**4**	4
pRMC2:*vgaA*$_{LC}$ ($R_{232}A$)	**16**	**2**	**8**	**2**	4

Table S3. Minimum inhibitory concentrations (MICs) of ribosome-targeting antibiotics against *L. monocytogenes* EGD-e expressing VgaL (Lmo0919). 5 x 10^5 CFU/mL (approximately OD_{600} 0.0003) of *L. monocytogenes EGDe*, Δ*lmo0919* (markerless) strain with integrated empty pIMK3 plasmid, or with pIMK3 encoding VgaL or VgaL-HTF was used to inoculate BHI media supplemented with 50 µg/mL kanamycin to maintain the integrative pIMK3 plasmid, 1 mM IPTG to induce expression of VgaL as well as increasing concentrations of antibiotics. After 16–20 hours at 37 °C without shaking, the presence or absence of bacterial growth was scored by eye. The MIC values that exceed the empty vector control lacking chromosomal *lmo0919* are shown in bold.

		MIC, µg/mL			
antibiotic class	antibiotic	*L. monocytogenes* EGDe::pIMK3	*L. monocytogenes* EGDe::Δ*lmo0919* pIMK3	*L. monocytogenes* EGDe::Δ*lmo0919* pIMK3 *vgaL*	*L. monocytogenes* EGDe::Δ*lmo0919* pIMK3 *vgaL-HTF*
phenicols	chloramphenicol	4	4	4	4
oxazolidinones	linezolid	1	1	1	
macrolides	erythromycin	< 0.125	< 0.125	< 0.125	< 0.125
lincosamides	lincomycin	**2**	0.5	**4**	**4**
pleuromutilins	tiamulin	**16-32**	0.125	**32**	**16-32**
streptogramins	virginiamycin M1	**32**	4-8	**64**	**32**
	virginiamycin S1	1	1	1	1
tetracyclines	tetracycline	0.25	0.25	0.25	0.25

Table S4. Cryo-EM data collection, modelling and refinement statistics.

	LsaA-70S (EMDB-12331) (PDB 7NHK)	VgaA$_{LC}$-70S (EMDB-12332) (PDB 7NHL)	*S. aureus* 70S (EMDB-12333) (PDB 7NHM)	VgaL-70S (EMDB-12334) (PDB 7NHN)
**Data collection and processing**				
Magnification	130 000	165 000	165 000	165 000
Voltage (kV)	300	300	300	300
Electron exposure ($e^{-}/Å^2$)	38.0	26.3	26.3	28.28
Defocus range (μm)	−0.7–2.2	−0.7–1.9	−0.7–1.9	−0.8–2.0
Pixel size (Å)	1.041	0.82	0.82	0.82
Symmetry imposed	None	None	None	None
Initial particle images (no.)	61 009	165 827	165 827	83 340
Final particle images (no.)	59 262	35 129	61 910	45 548
Map resolution (Å)	2.9	3.1	3.1	2.9
FSC threshold	0.143	0.143	0.143	0.143
**Refinement**				
Map sharpening *B* factor ($Å^2$)	−35.42	−62.31	−56.43	−68.16
Model composition				
Non-hydrogen atoms	146 171	145 988	140 264	144 398
Protein residues	5 938	5 839	5 388	5 715
RNA residues	4 623	4 647	4 554	4 617
R.m.s. deviations				
Bond lengths (Å)	0.007	0.012	0.012	0.012
Bond angles (°)	0.895	1.095	1.113	1.108
Validation				
MolProbity score	1.51	1.67	1.58	1.53
Clashscore	3.03	3.42	2.94	3.12
Poor rotamers (%)	0.04	0.10	0.13	0.00
Ramachandran plot				
Favored (%)	93.64	90.47	91.85	93.42
Allowed (%)	6.36	9.53	8.13	6.58
Disallowed (%)	0.0	0.0	0.02	0.0

Table S5. Strains and Plasmids used in this study. Plasmid and strain construction is described in detail in supplemental text. *Denotes a plasmid constructed by the PEP facility at Umeå University.

Strain	Description	Source
L. monocytogenes EGDe	Wild-type serotype 1/2a strain	Glaser *et al.*, 2001[12]
L. monocytogenes EGDe::pIMK3	EGDe with empty pIMK3 plasmid containing P_{help} promoter integrated at $tRNA^{Arg}$ locus	This work
L. monocytogenes EGDe::pIMK3*lmo0919*^{HTF}	EGDe with VgaL-HTF overexpressed from the P_{help} promoter integrated at $tRNA^{Arg}$ locus	This work
L. monocytogenes EGDe::pIMK3*lmo0919*$^{EQ2\text{-}HTF}$	EGDe with VgaL EQ2-HTF overexpressed from the P_{help} promoter integrated at $tRNA^{Arg}$ locus	This work
L. monocytogenes EGDe::Δ*lmo0919*	EGDe harboring a *lmo0919* marker less deletion lacking VgaL	This work
L. monocytogenes EGDe::Δ*lmo0919*::pIMK3	EGDe::Δ*lmo0919* with empty pIMK3 plasmid containing P_{help} promoter integrated at $tRNA^{Arg}$ locus	This work
L. monocytogenes EGDe::Δ*lmo0919*::pIMK3*lmo0919*	EGDe::Δ*lmo0919* with VgaL overexpressed from the P_{help} promoter integrated at $tRNA^{Arg}$ locus	This work
L. monocytogenes EGDe::Δ*lmo0919*::pIMK3*lmo0919*^{HTF}	EGDe::Δ*lmo0919* with VgaL-HTF overexpressed from the P_{help} promoter integrated at $tRNA^{Arg}$ locus	This work
L. monocytogenes EGDe:: Δ*lmo0919*::pIMK3*lmo0919*$^{HFT\text{-}EQ2}$	EGDe::Δ*lmo0919* with VgaL-EQ2-HTF overexpressed from the P_{help} promoter integrated at $tRNA^{Arg}$ locus	This work
E. faecalis OG1RF	Rif^r Fus^r; WT *E. faecalis*	Singh *et al.*, 2002[13]
E. faecalis TX5332	Rif^r Fus^r Kan^r; *lsa* gene disruption mutant (OG1RF *lsa*::pTEX4577)	Davis *et al.*, 2001[14]
S. aureus SH1000	Functional *rsbU*$^+$ derivative of *S. aureus* 8325-4	O'Neill, 2001, and Horsburgh *et al.*, 2002 [15, 16]
E. coli S17.1	*E. coli* strain used for conjugative plasmid transfer to *L. monocytogenes*	Simon *et al.*, 1983[17]

Plasmid	Description	Reference
pIMK3	Kan^r; Listerial tRNA^{Arg} locus specific integrative vector for high-level IPTG-induced protein expression from the P_{help} promoter	Monk *et al.,* 2008[18]
pMAD	Amp^r, Ery^r; *lacZ*; thermosensitive shuttle vector used for allelic exchange in *L. monocytogenes*	Arnaud *et al.*, 2004[19]
pHT009	Amp^r, Km^r; thrC locus specific integrative vector for high-level IPTG-induced protein expression from the $P_{hy\text{-}spnak}$ promoter	Crowe-McAuliffe *et al.*, 2018[7]
VHp689	pMAD Δ*lmo0919*	This work
VHp690	pIMK3:*lmo0919*	This work
VHp692	pIMK3:*lmo0919-HTF*	This work
VHp693	pIMK3:*lmo0919-EQ2-HTF*	This work
pTX5333	Cm^r; *E. faecalis-E. coli* shuttle plasmid expressing LsaA from native promoter	Singh *et al.*, 2002[13]
pCIE	Cm^r; *E. faecalis-E. coli* shuttle plasmid for cCF10 induced expression of proteins	Weaver *et al.*, 2017[20]
VHp100	pCIE:*lsaA-HTF*	This work*
VHp149	pCIE:*lsaA-EQ2-HTF*	This work*
VHp369	pHT009-*lsaA*	This work
VHp426	pCIE, Sc^r; Cm^r gene swapped to spectinomycin resistance (Sc^r) gene	This work*
VHp431	VHp426:*lsa*	This work*
VHp526	pHT009-*lsaA*K244A	This work
VHp526	pHT009-*lsaA*K244A	This work
pRMC2	Amp^r, Cm^r; *E. coli - S. aureus* shuttle plasmid for tetracycline-regulable expression of proteins in the latter host.	Corrigan *et al.*, 2009[21]
pRMC2:*vgaA-FLAG$_3$*	pRMC2 expressing C-terminally $FLAG_3$ tagged $VgaA_{LC}$	This work
pRMC2:*vgaA-EQ2-FLAG$_3$*	pRMC2 expressing C-terminally $FLAG_3$ tagged $VgaA_{LC}$-$E_{105}Q$, $E_{410}Q$	This work
pRMC2:*$vgaA_{LC}$*	pRMC2 expressing wild-type $VgaA_{LC}$	This work
pRMC2:*$vgaA_{LC}$* ($K_{208}A$)	pRMC2 expressing $VgaA_{LC}$ ^{K208A}	This work
pRMC2:*$vgaA_{LC}$* ($S_{211}A$)	pRMC2 expressing $VgaA_{LC}$ ^{S211A}	This work
pRMC2:*$vgaA_{LC}$* ($S_{212}A$)	pRMC2 expressing $VgaA_{LC}$ ^{S212A}	This work
pRMC2:*$vgaA_{LC}$* ($S_{213}A$)	pRMC2 expressing $VgaA_{LC}$ ^{S213A}	This work
pRMC2:*$vgaA_{LC}$* ($K_{216}A$)	pRMC2 expressing $VgaA_{LC}$ ^{K216A}	This work
pRMC2:*$vgaA_{LC}$* ($K_{218}A$)	pRMC2 expressing $VgaA_{LC}$ ^{K218A}	This work
pRMC2:*$vgaA_{LC}$* ($V_{219}A$)	pRMC2 expressing $VgaA_{LC}$ ^{V219A}	This work
pRMC2:*$vgaA_{LC}$* ($Y_{223}A$)	pRMC2 expressing $VgaA_{LC}$ ^{Y223A}	This work
pRMC2:*$vgaA_{LC}$* ($F_{224}A$)	pRMC2 expressing $VgaA_{LC}$ ^{F224A}	This work
pRMC2:*$vgaA_{LC}$* ($S_{226}A$)	pRMC2 expressing $VgaA_{LC}$ ^{S226A}	This work
pRMC2:*$vgaA_{LC}$* ($K_{227}A$)	pRMC2 expressing $VgaA_{LC}$ ^{K227A}	This work
pRMC2:*$vgaA_{LC}$* ($Q_{228}A$)	pRMC2 expressing $VgaA_{LC}$ ^{Q228A}	This work

pRMC2:*vgaA*$_{LC}$ (K$_{229}$A)	pRMC2 expressing VgaA$_{LC}$ ^{K229A}	This work
pRMC2:*vgaA*$_{LC}$ (K$_{230}$A)	pRMC2 expressing VgaA$_{LC}$ ^{K230A}	This work
pRMC2:*vgaA*$_{LC}$ (A$_{232}$A)	pRMC2 expressing VgaA$_{LC}$ ^{A232A}	This work

Table S6 Primers used in this study.

Name	Sequence
vgaA$_{LC}$-F	GGTGGTGGTACCAGGATGAGGAAATATGAAAA
vgaA$_{LC}$-R	GGTGGTGAATTCGGTAATTTATTTATCTAAATTTCTT
VHKT12	CCCCCCATGGCATCTACAATCGAAATAAATC
VHKT13	GGGGCTGCAGTTAGCCTTTGTCATCGTC
VHKT14	AGACAGCAATTTAGTGGCGGCCATCATCATCATC
VHKT15	ATGATGATGGCCGCCACTAAATTGCTGTCTTTTTG
VKT35	GGGGGGATCCATCACTAGCCGAATCCAAAC
VKT36	GGGGGAATTCAAAAAATAACCTCCTGAATATTTTCAGAG
VHKT37	GGGGGAATTCAAAAAATAACCTCCTGAATATTTTCAGAG
VHKT38	GGGGCCATGGCGTGCTGTACGGTATGC
VHKT39	GGGGCTGCAGTTAACTAAATTGCTGTCTTTTTG
VHT123	CATTATCGCTCTCTCCTTCGTCGACTAAGCTAATTG
VHT125	TAAGCATGCAAGCTAATTCGGTGGAAACGAGG
VHT127	CGACGAAGGAGAGAGCGATAATGTCGAAAATTGAACTAAAACAACTATC
VHT128	CACCGAATTAGCTTGCATGCTTATGATTTCAAGACAATTTTTTTATCTGTTA
VHT264	AGCAGACCAACCAACAAGCAATCTTGATGTCG
VHT265	TGGTTGGTTGATCAAGAATCAAGAAATTGGCGT
VHT266	TCTTGATCAACCAACCAACTATTTGGATATCTACGCAATGGAA
VHT267	TTGTTGGTTGGTCTGCTAGGAGAACACTTGGATTTTGGCGCA
VHP303	GCATCACCTTCACGGTTCATCGACCATTCCGCT
VHP304	GTACGGCAACGCTAAGGAAAAAGGGAGCGGGGCGA
VHP305	CAATCGCCCCGCTCCCTTTTTCCTTAGCGT
VHP306	CGGATACAGGAGCCATTGGTGCCCGGGCA

Supplementary References

1. Johnson ZL, Chen J. ATP binding enables substrate release from Multidrug Resistance Protein 1. *Cell* **172**, 81-89 (2018).

2. Heuer A*, et al.* Structure of the 40S–ABCE1 post-splitting complex in ribosome recycling and translation initiation. *Nat Struct Mol Biol* **24**, 453-460 (2017).

3. Becker T*, et al.* Structural basis of highly conserved ribosome recycling in eukaryotes and archaea. *Nature* **482**, 501-506 (2012).

4. Voorhees RM, Schmeing TM, Kelley AC, Ramakrishnan V. The mechanism for activation of GTP hydrolysis on the ribosome. *Science* **330**, 835-838 (2010).

5. Matzov D*, et al.* Structural insights of lincosamides targeting the ribosome of *Staphylococcus aureus*. *Nucleic Acids Res* **45**, 10284-10292 (2017).

6. Polikanov YS, Steitz TA, Innis CA. A proton wire to couple aminoacyl-tRNA accommodation and peptide-bond formation on the ribosome. *Nat Struct Mol Biol* **21**, 787-793 (2014).

7. Crowe-McAuliffe C*, et al.* Structural basis for antibiotic resistance mediated by the *Bacillus subtilis* ABCF ATPase VmlR. *Proc Natl Acad Sci U S A* **115**, 8978-8983 (2018).

8. Su W*, et al.* Ribosome protection by antibiotic resistance ATP-binding cassette protein. *Proc Natl Acad Sci U S A* **115**, 5157-5162 (2018).

9. Su T*, et al.* Structure and function of Vms1 and Arb1 in RQC and mitochondrial proteome homeostasis. *Nature* **570**, 538-542 (2019).

10. Schlünzen F, Pyetan E, Fucini P, Yonath A, Harms JM. Inhibition of peptide bond formation by pleuromutilins: the structure of the 50S ribosomal subunit from *Deinococcus radiodurans* in complex with tiamulin. *Mol Microbiol* **54**, 1287-1294 (2004).

11. Noeske J, Huang J, Olivier NB, Giacobbe RA, Zambrowski M, Cate JH. Synergy of streptogramin antibiotics occurs independently of their effects on translation. *Antimicrob Agents Chemother* **58**, 5269-5279 (2014).

12. Glaser P, *et al.* Comparative genomics of *Listeria* species. *Science* **294**, 849-852 (2001).

13. Singh KV, Weinstock GM, Murray BE. An Enterococcus faecalis ABC homologue (Lsa) is required for the resistance of this species to clindamycin and quinupristin-dalfopristin. *Antimicrob Agents Chemother* **46**, 1845-1850 (2002).

14. Davis Dv*, et al. Enterococcus faecalis* multi-drug resistance transporters: application for antibiotic discovery. *J Mol Microbiol Biotechnol* **3**, 179-184 (2001).

15. O'Neill A. *Staphylococcus aureus* SH1000 and 8325-4: comparative genome sequences of key laboratory strains in staphylococcal research. *Lett Appl Microbiol* **51**, 358-361 (2010).

16. Horsburgh MJ, Aish JL, White IJ, Shaw L, Lithgow JK, Foster SJ. σB modulates virulence determinant expression and stress resistance: characterization of a functional *rsbU* strain derived from *Staphylococcus aureus* 8325-4. *J Bacteriol* **184**, 5457-5467 (2002).

17. Simon R, Priefer U, Pühler A. A broad host range mobilization system for *in vivo* genetic engineering: transposon mutagenesis in Gram negative bacteria. *Bio/technology* **1**, 784-791 (1983).

18. Monk IR, Gahan CG, Hill C. Tools for functional postgenomic analysis of *Listeria monocytogenes*. *Appl Environ Microbiol* **74**, 3921-3934 (2008).

19. Arnaud M, Chastanet A, Débarbouillé M. New vector for efficient allelic replacement in naturally nontransformable, low-GC-content, gram-positive bacteria. *Appl Environ Microbiol* **70**, 6887-6891 (2004).

20. Weaver KE*, et al.* Examination of *Enterococcus faecalis* toxin-antitoxin system toxin Fst function utilizing a pheromone-inducible expression vector with tight repression and broad dynamic range. *J Bacteriol* **199**, e00065-00017 (2017).

21. Corrigan RM, Foster TJ. An improved tetracycline-inducible expression vector for *Staphylococcus aureus*. *Plasmid* **61**, 126-129 (2009).
