# Supplementary material for: Structural basis of ABCF-mediated resistance to pleuromutilin, lincosamide, and streptogramin A antibiotics in Gram-positive pathogens
Source: Nat Commun. 2021 Jun 11;12:3577. doi: 10.1038/s41467-021-23753-1 (PMC8196190; doi:10.1038/s41467-021-23753-1)
Supplement: Supplementary file 3 — Reporting Summary [file 41467_2021_23753_MOESM3_ESM.pdf]

nature research

Corresponding author(s): Daniel Wilson and Vasili Hauryliuk

Last updated by author(s): May 4, 2021

Reporting Summary

Nature Research wishes to improve the reproducibility of the work that we publish. This form provides structure for consistency and transparency in reporting. For further information on Nature Research policies, see our Editorial Policies and the Editorial Policy Checklist.

Statistics

For all statistical analyses, confirm that the following items are present in the figure legend, table legend, main text, or Methods section.

n/a	Confirmed	
☐	☒	The exact sample size (*n*) for each experimental group/condition, given as a discrete number and unit of measurement
☐	☒	A statement on whether measurements were taken from distinct samples or whether the same sample was measured repeatedly
☐	☒	The statistical test(s) used AND whether they are one- or two-sided<br>*Only common tests should be described solely by name; describe more complex techniques in the Methods section.*
☒	☐	A description of all covariates tested
☒	☐	A description of any assumptions or corrections, such as tests of normality and adjustment for multiple comparisons
☐	☒	A full description of the statistical parameters including central tendency (e.g. means) or other basic estimates (e.g. regression coefficient) AND variation (e.g. standard deviation) or associated estimates of uncertainty (e.g. confidence intervals)
☒	☐	For null hypothesis testing, the test statistic (e.g. *F*, *t*, *r*) with confidence intervals, effect sizes, degrees of freedom and *P* value noted<br>*Give P values as exact values whenever suitable.*
☒	☐	For Bayesian analysis, information on the choice of priors and Markov chain Monte Carlo settings
☒	☐	For hierarchical and complex designs, identification of the appropriate level for tests and full reporting of outcomes
☒	☐	Estimates of effect sizes (e.g. Cohen's *d*, Pearson's *r*), indicating how they were calculated

Our web collection on statistics for biologists contains articles on many of the points above.

Software and code

Policy information about availability of computer code

Data collection	CryoEM data were collected using the EPU software version 2.8 (FEI, Netherlands)
Data analysis	Sequence alignment and phylogenetic analysis: Sequences were aligned with Clustal Omega v1.2.3 and edited by hand to match the structures. Cryo-EM: RELION v3.0 and v3.1 with MotionCor2 v1.2.1, Gctf v1.06, and Gautomatch v0.56 for processing micrographs, picking particles, classification and refining cryo-EM maps. ResMap v1.1.4 for calculating local resolution and SPHIRE (SPARX v4.0) for filtering according to local resolution. Coot v0.8 and v0.92 for model building and Phenix (dev-2947-000) for model refinement and statistics. Figures were generated using Pymol v2.4, Chimera v1.14, and ChimeraX v1.0.

For manuscripts utilizing custom algorithms or software that are central to the research but not yet described in published literature, software must be made available to editors and reviewers. We strongly encourage code deposition in a community repository (e.g. GitHub). See the Nature Research guidelines for submitting code & software for further information.

Data

Policy information about availability of data

All manuscripts must include a data availability statement. This statement should provide the following information, where applicable:

- Accession codes, unique identifiers, or web links for publicly available datasets
- A list of figures that have associated raw data
- A description of any restrictions on data availability

The cryo-electron microscopy maps and the respective coordinates for electron-microscopy-based model have been deposited in the EMDataBank, PDB, and EMPIAR under the accession numbers EMD-12331, EMD-12332, EMD-12333 and EMD-12334, PDB ID 7NHK, PDB ID 7NHL, PDB ID 7NHM, and PDB ID 7NHN. Micrographs have been deposited in EMPIAR with accession numbers EMPIAR-10682, EMPIAR-10683, and EMPIAR-10684.
tRNA microarrays: Data have been deposited in Gene Expression Omnibus (GEO) database under accession GSE162168.

Field-specific reporting

Please select the one below that is the best fit for your research. If you are not sure, read the appropriate sections before making your selection.

☒ Life sciences ☐ Behavioural & social sciences ☐ Ecological, evolutionary & environmental sciences

For a reference copy of the document with all sections, see nature.com/documents/nr-reporting-summary-flat.pdf

Life sciences study design

All studies must disclose on these points even when the disclosure is negative.

Sample size	tRNA microarrays were performed in duplicate as per convention.
Data exclusions	Micrographs with low estimated resolution or poorly fitted CTFs were excluded from further processing, as were particles that clustered into poorly defined classes during 2D and 3D classification.
Replication	For tRNA microarrays, two immunoprecipitation samples were measured against a single lysate and replications were successful as shown in Figure 2e.
Randomization	For 3D refinement in RELION, particles are randomly placed in one of two subsets. These subsets are maintained for CTF refinement. Otherwise, no randomization was performed.
Blinding	No blinding was performed as blinding is not possible or not applicable for the experiments.

Reporting for specific materials, systems and methods

We require information from authors about some types of materials, experimental systems and methods used in many studies. Here, indicate whether each material, system or method listed is relevant to your study. If you are not sure if a list item applies to your research, read the appropriate section before selecting a response.

Materials & experimental systems

n/a	Involved in the study
☐	☒ Antibodies
☒	☐ Eukaryotic cell lines
☒	☐ Palaeontology and archaeology
☒	☐ Animals and other organisms
☒	☐ Human research participants
☒	☐ Clinical data
☒	☐ Dual use research of concern

Methods

n/a	Involved in the study
☒	☐ ChIP-seq
☒	☐ Flow cytometry
☒	☐ MRI-based neuroimaging

Antibodies

Antibodies used	F3165 Sigma-Aldrich Monoclonal ANTI-FLAG® M2 antibody produced in mouse<br>clone M2, purified immunoglobulin (Purified IgG1 subclass), buffered aqueous solution (10 mM sodium phosphate, 150 mM NaCl, pH 7.4, containing 0.02% sodium azide)
Validation	https://api.sigmaaldrich.com/deepweb/assets/sigmaaldrich/quality/spec/332/602/F3165-BULK________SIGMA____.pdf
